# Active Iterative Social Inference in Multi-Trial Signaling Games

**DOI:** 10.1162/opmi_a_00074

**Published:** 2023-04-05

**Authors:** Asya Achimova, Gregory Scontras, Ella Eisemann, Martin V. Butz

**Affiliations:** Research Training Group 1808 “Ambiguity: Production and Perception”, University of Tübingen, Tübingen, Germany; Department of General and Computational Linguistics, University of Tübingen, Tübingen, Germany; Department of Language Science, University of California, Irvine, USA; Institute of Vocational Education and Work Studies, Technische Universität Berlin, Berlin, Germany; Department of Computer Science and Department of Psychology, University of Tübingen, Tübingen, Germany

**Keywords:** pragmatics, social learning, sequential learning, ambiguity, online experiments, inference, learning about others

## Abstract

Human behavioral choices can reveal intrinsic and extrinsic decision-influencing factors. We investigate the inference of choice priors in situations of referential ambiguity. In particular, we use the scenario of signaling games and investigate to which extent study participants profit from actively engaging in the task. Previous work has revealed that speakers are able to infer listeners’ choice priors upon observing ambiguity resolution. However, it was also shown that only a small group of participants was able to strategically construct ambiguous situations to create learning opportunities. This paper sets to address how prior inference unfolds in more complex learning scenarios. In Experiment 1, we examine whether participants accumulate evidence about inferred choice priors across a series of four consecutive trials. Despite the intuitive simplicity of the task, information integration turns out to be only partially successful. Integration errors result from a variety of sources, including transitivity failure and recency bias. In Experiment 2, we investigate how the ability to actively construct learning scenarios affects the success of prior inference and whether the iterative settings improve the ability to choose utterances strategically. The results suggest that full task engagement and explicit access to the reasoning pipeline facilitates the invocation of optimal utterance choices as well as the accurate inference of listeners’ choice priors.

## INTRODUCTION

With an objective in mind but multiple options at hand, an agent must make a choice about the appropriate action to take. When observing such choices, we can learn about the mental states of the agents who made them: what led the agent to choose option *a* over options *b* or *c*? The current paper explores a particular type of social scenario that presents choices to an agent: cases of referential ambiguity where one particular referent must be chosen in response to an ambiguous utterance, which opens up multiple choice options. In this process, listeners rely on their choice priors—the beliefs, preferences, or desires that shape an agent’s choice behavior—as well as a variety of pragmatic reasoning strategies, to come to a decision. We explore how people reason about the apparent choice priors of their social partners as they resolve ambiguity, particularly in cases where the speaker can create ambiguous situations actively and iteratively over several interaction trials.

The human ability to interpret each other’s behavior as driven by motives, intentions, and goals is a critical component of Theory of Mind. Early work in this direction developed within the attribution theory (Jones & Davis, 1965; Kelley, 1967; Kelley & Stahelski, 1970). The ability to infer mental states of others upon observing their behavioral choices develops early in life. Infants as young as 18 months of age have been shown to infer the preferences of the experimenter in a setup where the experimenter is pulling toys from buckets, and the buckets differ in their distributions of types of toys (Kushnir et al., 2010). In a different set of experiments, this time with adults, Baker et al. (2017) show that participants are able to infer the food preferences of an agent upon observing how the agent navigates the space between several food trucks. The authors furthermore model the inference process as Bayesian Theory of Mind inference. Jara-Ettinger et al. (2016, 2020) argue that this social inference is an integral part of a naive utility calculus—an intuitive theory humans have about other agents making choices. Here, we explore potential benefits of actively engaging the agent, who makes social inferences iteratively across four trials of distinct signaling game interactions, by enabling her to actively choose utterances in each trial. The utterances selectively restrict the response choices available to the listener. We further embed our task in a 4-trial learning scenario where participants observe the behavior of a particular simulated agent through several iterations.

Iterative decision-making has been previously explored with computational models of social inference (Evans et al., 2016; Jara-Ettinger et al., 2020). Integrating information across a sequence of trials entails not only retaining information in memory over a period of time longer than a single trial, but also performing additional inference steps. For example, participants may need to perform transitive inferences in a given learning scenario, inferring that *a* is rated higher than *c* upon observing *a* > *b* and *b* > *c* scenarios. Ciranka et al. (2022) investigated how inference success depends on the type of feedback provided to the participants. They contrasted a model where full feedback is provided and participants do not have to make transitive inferences about the ordering of values (Bryant & Trabasso, 1971; Wynne, 1995) with a partial feedback model. In the full feedback model, if *a* is chosen over *b*, the model increases the value of *a* and decreases the value of *b* at the same rate. In the partial feedback model, on the contrary, the implicit value update is asymmetric: the model only increases or decreases the value of the chosen or discarded property, respectively, but does not both increase and decrease values. The authors demonstrated that transitive inference can be efficiently modeled as a reinforcement learning scenario and demonstrated that the model gives correct predictions for a range of cognitive effects reported in psychophysics and decision-making.

From a reinforcement learning (RL) perspective, preference inference can be modeled as a particular instance of hidden value learning (Sutton & Barto, 2018), inverse RL (Hadfield-Menell et al., 2016), or inverse decision-making (Jern et al., 2017). Jern et al. (2017) investigate how participants infer preferences of agents choosing objects with multiple attributes. Building on the naive utility calculus model of Jara-Ettinger et al. (2016), the authors offer an inverse decision-making model that accounts for human inferences. Their model relies on a decision-making function that provides an explicit link between the preferences of an agent and a decision that she makes. Still, the choices humans make can be motivated by a multitude of factors and precisely specifying which of them drive decision making is a complex task. For example, the model of Evans et al. (2016) infers not only preferences but also beliefs of the agent, motivated by the general Beliefs-Desires-Intention model (Bratman, 1984).

In our work, we will use preference inference as a test scenario to investigate how choice priors more generally can be inferred in situations where a participant makes a behavioral choice. Even though we ask participants to infer potential “preferences” of a simulated listener, due to the abstractness of the task, we cannot specifically test whether it is the preferences or other factors that determine the choices of objects in the task. Rather, we regard the inference of choice priors in our scenarios as a form of social inference concentrating on the following aspects. First, we investigate whether participants successfully integrate information iteratively across the four trials. Second, we explore how an active role of the participant in the learning scenario affects inference success. In this work, we focus on the empirical investigation of choice priors, without any further differentiation of the factors that contribute to them. The results imply that the active creation of choice options helps improving the social inference of choice priors.

## AMBIGUITY RESOLUTION PARADIGM

We use a signaling game scenario in which choices can be made and reasoned about. In classic signaling games, a speaker makes an utterance and signals an object to the listener (Lewis, 1975). The listener’s task is to identify the intended object. Typically, signaling games are used to investigate how speakers make utterance choices to maximize the chance that the listener will choose the target object. Moreover, the listeners’ choice behavior has been investigated in situations when the utterance applies to more than one object—a case of referential ambiguity (e.g., Frank & Goodman, 2012; Franke & Jäger, 2016; Goodman & Frank, 2016). In contrast, here we focus on the extent to which speakers can draw iterative social inferences about the behavioral choice priors of listeners. Speakers observe listener’s object choices in four successive signal game interaction trials. In each trial, a particular set of three objects is shown, a particular utterance is provided, and the consequent object choice is indicated. Participants, acting as the speaker, are then asked to infer the apparent choice priors of the listener (instructed as “apparent preference”). Moreover, in the second experiment, speakers are additionally asked to choose the potentially choice-restricting utterance.

**Figure 1.**
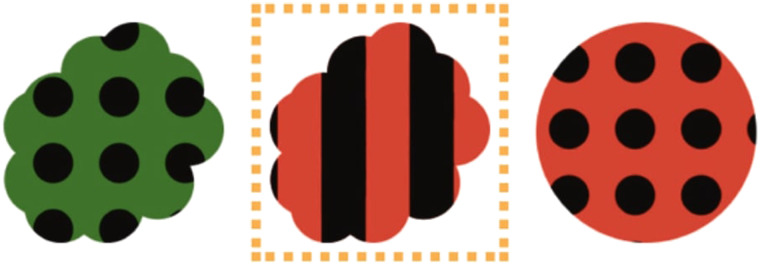
**Example preference-inference communication scenario from Achimova et al. (2022).** Participants see the three-object scenario, observe that a speaker produced an utterance (e.g., “red”) as an instructive choice request, and are informed about the listener’s consequent choice (i.e., picking the striped red cloud, as indicated by the orange dotted square).

The general potential of such signaling game scenarios to infer listeners’ choice priors has been previously explored in Achimova et al. (2022). The authors have shown that participants were indeed able to infer listener priors upon observing the listener’s choice of an object given an ambiguous object choice request. For example, in Figure 1, participants might observe that the speaker said “red”—a referentially-ambiguous utterance that is consistent with either of the two red objects—and the listener resolves the ambiguity by choosing the center object, as indicated by the orange square. The task was to decide which “preferences” the listener may have used to make her choice. What Achimova et al. (2022) labelled “preferences” operationalized as choice priors over potential object selections in their Bayesian model. Accordingly, we refer to the more general term “choice prior” in the remainder of the current paper. In response to a scenario like Figure 1, participants were more likely to conclude that the listener has larger choice priors for clouds and stripes than for circles and polka-dotted objects.

**Figure 2.**
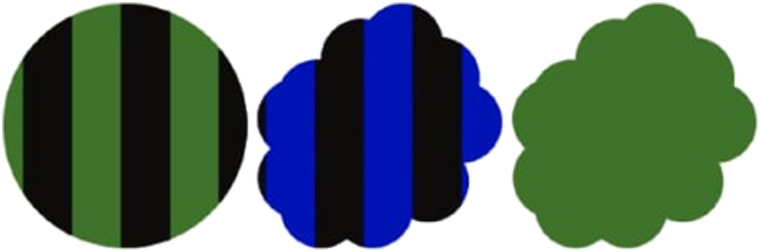
**Exemplar utterance-choice communication scenario from Achimova et al. (2022).** In a typical utterance choice task, participants are asked to use an object property (e.g., “blue,” “green,” “cloud,” etc.) as a choice instruction to the listener, such that they can expect to learn about the choice priors of the listeners when observing their consequent object choice. As a result, ambiguous utterances typically promise more information gain than unambiguous ones.

For strategically creating cases of ambiguity, Achimova et al. (2022) had participants help the speaker select their utterances in an effort to better learn about the listener’s choice priors. So, for example, when confronted with the scenario in Figure 2, a subset of participants would suggest “green”, “striped”, or “cloud” rather than “circle”, “blue”, or “solid” – because these utterances create a referential ambiguity that can reveal information about listeners’ choice priors upon observing their object choice. Surprisingly, a varying but significant subset of other participants systematically selected *un*-ambiguous utterances, failing to pursue information gain about choice priors but preferring ambiguity avoidance.^1^

Achimova et al. (2022) articulate a hypothesis about how speakers reason about choice priors in the context of ambiguity—a hypothesis in the form a computational cognitive model formulated within the Rational Speech Act modeling framework (Goodman & Frank, 2016). While the authors found support for their hypothesis in terms of the model’s ability to quantitatively predict human behavior in the experimental tasks, the model makes an interesting—and as yet untested—prediction: when observing multiple ambiguity resolution trials, participants should be able to gain even deeper insights into the (potentially complex) choice priors that the listener may use to resolve cases of ambiguity. We explore this expectation in the current work. In particular, we expected that participants will be able to both integrate gained knowledge over subsequent trials and choose ambiguous utterances in a more strategic manner when in a multi-trial setting. Moreover, we expected that the participants that choose maximally effective ambiguous utterances will also learn more from the consequent ambiguity resolution behavior.

To test these expectations, we asked to what extent participants can learn a more complex hierarchy of choice priors when experiencing four subsequent signaling game interaction trials. Moreover, we asked whether participants’ inference success could benefit from enabling active utterance choices. Over the course of two experiments, we show that (i) multi-trial learning about choice priors is possible (Exp. 1 & 2), (ii) the inference process suffers from a recency bias (Exp. 1 & 2), (iii) some participants manage to actively choose ambiguous utterances in search of information gain about choice priors (Exp. 2), and (iv) participants indeed learn more about the listeners’ choice priors when they actively pursue ambiguous utterances (Exp. 2).

## EXPERIMENT 1: ITERATED PRIOR INFERENCE

First, we extend the information-foraging experimental set-up from Achimova et al. (2022) to a multi-trial setting, seeing whether participants are able to learn about the (potentially-complex) priors of conversation partners in the context of ambiguous utterances.^2^ Rather than the single-trial design of the Achimova et al.’s (2022) experiment, here participants are exposed to four trials’ worth of interpretation behavior.

### Material and Methods

#### Participants.

We collected data online using the Prolific crowd-sourcing platform. Participants received £1.3 as compensation, and the experiment lasted approximately 9 minutes (*mean* = 8.58 minutes, *median* = 7.71 minutes). The experimental protocol was approved by the Psychology Department Ethics Committee at the University of Tübingen. We collected data from 55 participants.

#### Design.

**Figure 3.**
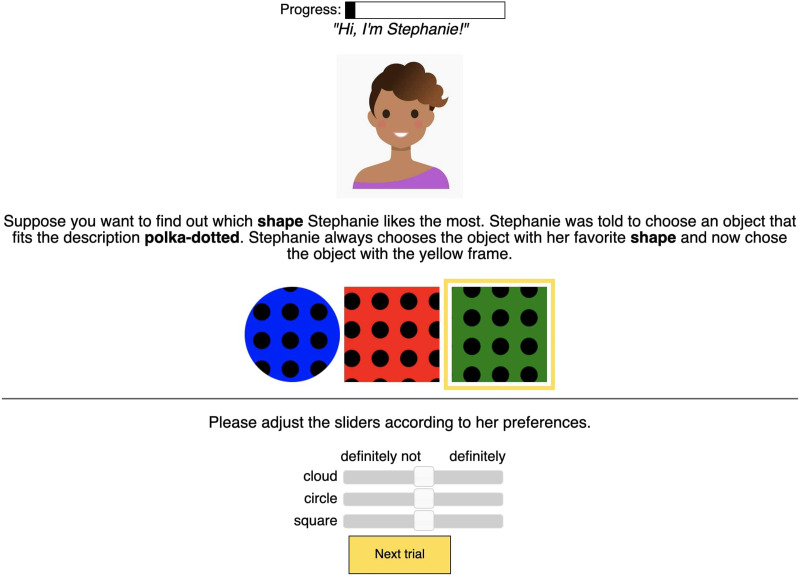
Sample trial for Experiment 1.

Participants completed 4 blocks of trials, each containing 4 trials. Within a block, we kept the simulated listener stable. Each listener had a name and an avatar. According to the test scenario, the listener picked an object that fit the description she heard, and she always picked her “favorite” shape, texture, or color (Figure 3). The task of the participant was to infer the preferences of the listener along a particular dimension: color, shape, or texture. To indicate the preferences, participants adjusted the sliders corresponding to the levels of the target property. For example, if a participant’s task was to infer shape preferences (as in Figure 3), she was asked to adjust the sliders “cloud”, “circle”, and “square”. At the end of the block, we provided feedback to the participants showing whether they inferred the preferences of the listener correctly. After that, participants proceeded to the next block.

The experiment featured two types of learning scenarios. In the *a* > *b* > *c* blocks, it was possible to learn the full preference hierarchy of the simulated listener upon observing their ambiguity resolution behavior. Over the course of four trials, participants saw scenarios that allowed learning that *a* is preferred over *b* and *b* is preferred over *c*. The *a* > *c* pair was never explicitly presented in the experiment, and thus participants were invited to make the transitivity inference themselves. Thus, if the task was to infer color preferences and the simulated listener preferred red over green and green over blue objects, critical trials showed the listener’s choice for each of these pairs. Partial hierarchy blocks, or *a* > *b*, *c* blocks, allowed participants to learn that one feature value was preferred to two other values, but there was no evidence for the relative preference of *b* and *c*. In other words, participants saw explicit evidence for both of the pairs *a* > *b* and *a* > *c*, but no evidence for the relationship between *b* and *c*.

Each block contained four trials: two critical ones and two fillers. Filler trials differed in their informativity. Redundant fillers provided the same information that was already presented in critical trials, offering additional evidence to the participants to test their hypotheses. Uninformative fillers featured scenarios where no learning about priors was possible. For instance, it is not possible to infer any preferences when the chosen utterance is unambiguous, as well when an utterance is ambiguous but it applies to objects that do not differ in their target feature value (e.g., the task is to infer color preferences, and the utterance “round” applies to 2 objects that are both red).

Thus, crossing two types of learning scenarios and two types of filler trials yielded four types of experimental blocks. Each participant completed all four blocks of trials; the block order was randomized.

### Results

#### Inference success.

**Figure 4.**
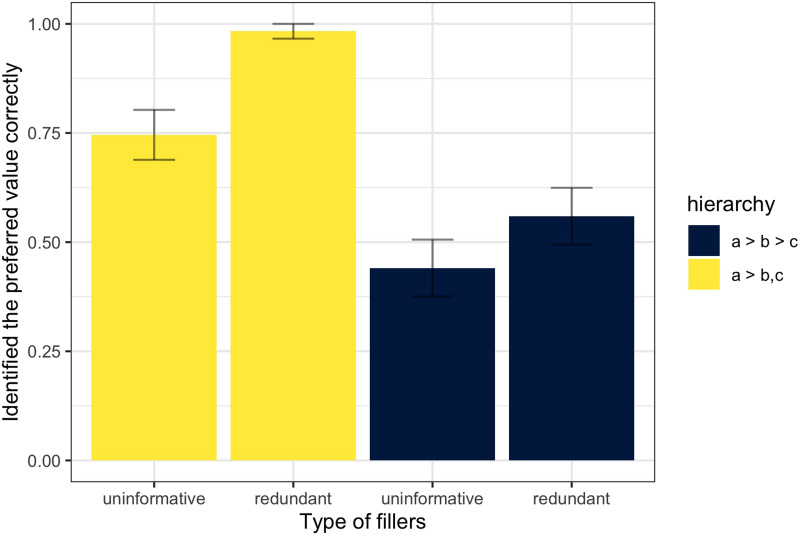
**Experiment 1: Proportion of blocks where the most preferred value has been identified correctly.** Learning success increases when redundant information is provided. Participants are less accurate when they infer priors in the *a* > *b* > *c* blocks compared to *a* > *b*, *c* blocks.

We begin presenting the results by identifying how often participants were able to infer the most preferred feature value (i.e., *a*) in different blocks of trials upon observing referential ambiguity resolution. Participants indicated the inferred preferences by adjusting slider values. To convert slider values into hierarchies, we simply ordered the slider inputs. If a participant assigned a value of 0.8 to *a*, 0.5 to *b*, and 0.1 to *c*, we recorded the inferred hierarchy as *a* > *b* > *c*. Thus, we evaluated for the last trial in each block whether a participant rated the property *a* higher than the properties *b* and *c*. Figure 4 plots success at inferring the preferred feature value by block type. The results of a generalized linear mixed effects model predicting preferred value inference by filler type (redundant vs. uniformative) and hierarchy (*a* > *b* > *c* vs. *a* > *b*, *c*) with random intercepts for participants demonstrates that participants were more successful in inferring the preferred value of the target feature in the simpler *a* > *b*, *c* blocks compared to the more complicated *a* > *b* > *c* blocks (*β* = 2.1892, *SE* = 0.397, *z* = 5.509, *p* < 0.001). Moreover, participants identified the correct preferred value less often when the fillers were uninformative compared to redundant fillers, since the latter provided confirmatory evidence (*β* = −1.132, *SE* = 0.352, *z* = −3.221, *p* < 0.01).

#### Integration of evidence across trials.

Our main question in Experiment 1 was whether participants were able to integrate the priors learned across a series of trials or whether they relied only on single trial evidence instead. For the first trial, the trial evidence and the available evidence are the same. However, for the second trial, the available evidence diverges from the trial evidence: the available evidence incorporates what could have been learned from the previous trials. Table 1 illustrates the difference between trial evidence and available evidence. Table 1 also provides examples of accumulated evidence, or the preference hierarchy indicated by a participant’s slider ratings on a given trial.

**Table 1.** Trial evidence vs. available evidence and the corresponding accumulated evidence score. True hierarchy: *a* > *b* > *c*

Trial	Trial evidence	Available evidence	Accumulated evidence	Accumulated evidence score
1	*a* > *b*	*a* > *b*	*a* > *b*	1
2	*b* > *c*	*a* > *b* > *c*	*a* > *b* > *c*	1

To assess the rates at which participants rely on evidence collected in previous trials, we first compared what relationship between the feature values *a*, *b*, and *c* the participants inferred (i.e., their accumulated evidence) and what relationship could in principle have been inferred given the set of trials a participant saw in that block (i.e., their available evidence). We assigned a value of 1 as their **accumulated evidence score** if a participant’s accumulated evidence matched the available evidence, suggesting that they successfully incorporated the information they previously learned; we assigned a value of 0 if a participant’s accumulated evidence did not match their available evidence, suggesting that they failed to integrate the evidence from the previous trial. Then, for each participant we calculated an average accumulated evidence score taking into account their performance either across all 16 trials (four blocks of four trials each) or across blocks with similar evidence type (i.e., *a* > *b* > *c* blocks vs. *a* > *b*, *c* blocks). This score reflects whether participants systematically integrated evidence throughout (portions of) the experiment. In addition, we also calculated the proportion of trials in which participants successfully inferred the priors just based on the information available in that trial. We refer to this metric as **trial evidence** and use it as a control showing task engagement.

**Figure 5.**
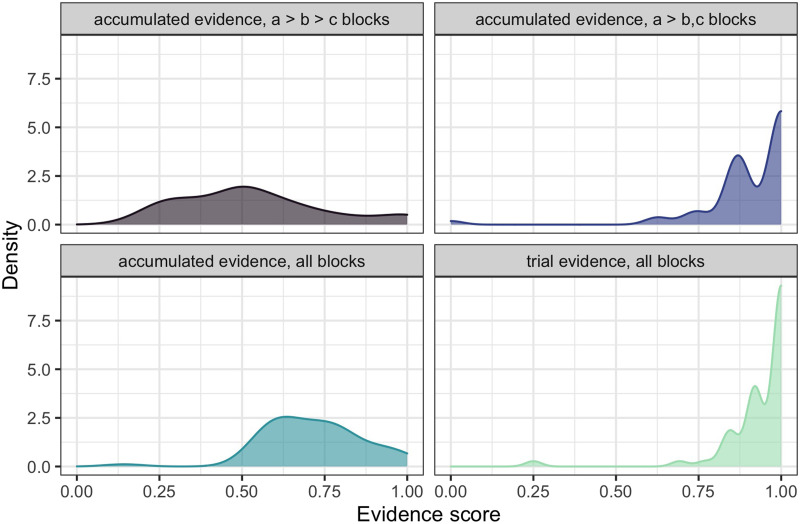
**Experiment 1: Density plots over the proportion of evidence-respecting preference inference trials, dependent on the available trial evidence (right bottom; average trial evidence score) or accumulated evidence (others; average accumulated evidence score) in all blocks (bottom) or block-respective (top).** While participants take the individual trial evidence well into account, more errors can be detected in the accumulated evidence and in particular in the trials where a more complex hierarchy (*a* > *b* > *c*) can be learned.

Figure 5 shows the distribution of participants’ accumulated evidence scores across different blocks of trials. The two upper panels contrast blocks where the full hierarchy (*a* > *b* > *c*) could have been learned vs. blocks where only partial information was available (*a* > *b*, *c*). The probability mass on the right side of each panel corresponds to participants who successfully integrated evidence. A linear mixed effects model analysis predicting the accumulated evidence scores (binomial variable) by block type confirms that participants were more successful at integrating evidence across the blocks of trials for the *a* > *b*, *c* blocks compared to *a* > *b* > *c* blocks (*β* = 0.375, *SE* = 0.028, *t* = 13.45, *p* < 0.001). This effect is expected: the partial hierarchy is cognitively simpler since the participants do not need to make any transitivity inferences and can simply rely on explicit evidence they register in a series of trials. Figure 5 also provides a more general measure of evidence accumulation success by looking at the performance in all blocks together (bottom left panel). This distribution is skewed to the right, suggesting that most of the participants did successfully accumulate evidence across a series of trials more than half of the time. This result confirms that prior inference upon observing ambiguity resolution extends to multi-trial scenarios. Finally, the bottom right panel of Figure 5 shows an example of a distribution when most participants achieve the highest score: trial evidence. Trial evidence concerns whether a participant uses the evidence available in a given trial on that trial. A high trial evidence score signals that participants paid attention throughout the experiment and performed the task as expected.

In sum, the distributions of trial evidence and accumulated evidence scores demonstrate that a) participants successfully infer the preferences of the simulated listener within a single trial (trial evidence); b) they integrate the inferred information across a series of trials (accumulated evidence); and c) they perform better in blocks with partial rather than full hierarchy available (block effect on accumulated evidence score). In the next subsection, we will take a closer look at those cases where participants fail to integrate evidence across trials.

#### Analysis of errors.

The performance on *a* > *b* > *c* blocks (upper left panel of Figure 5) shows that several participants made errors in accumulating evidence across trials. To better understand these errors, we ask whether the presentation order of trials affected their inference success: perhaps learning that *b* > *c* after learning that *a* > *c* made the transitivity inference that *a* > *c* more difficult. In order to assess participants’ inference success, we calculated the total inference score that participants achieved at the end of a block. The total inference score was calculated by assigning a value of 1 for every pair of the hierarchy identified correctly, namely *a* > *b*, *b* > *c*, and *a* > *c*, then summing over those values for the trials that made up a block. Table 2 shows several examples of scoring.

**Table 2.** Examples of total inference score calculation. True hierarchy: *a* > *b* > *c*

Inferred hierarchy	*a* > *b*	*b* > *c*	*a* > *c*	Total inference score
*a* > *b* > *c*	1	1	1	3
*a* > *b*, *c*	1	0	1	2
*b* > *a* > *c*	0	1	1	2
*c* > *a* > *b*	1	0	0	1
*c* > *b* > *a*	0	0	0	0

**Figure 6.**
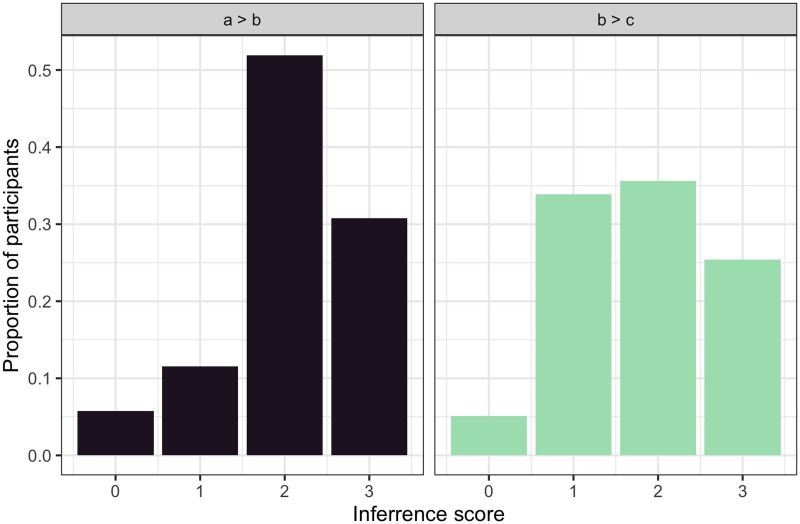
**Experiment 1: Normalized histograms over total inference scores for the blocks in which the full *a* > *b* > *c* preference hierarchy could be learned, dependent on whether the information about *a* > *b* (*left*) or *b* > *c* (*right*) was available in the last trial(s).** The total inference score counts the number of correctly ordered feature pairs in the inferred preferenc hierarchy. The results imply a tendency towards a recency bias: when information about *b* > *c* arrived last, participants generate more preference ordering errors (i.e., incorrectly ranking *b* over *a*).

We can now scrutinize the performance in *a* > *b* > *c* blocks by looking at the effect of trial order on the total inference scores. To be more precise, we are interested in the effect of early vs. late presentation of evidence about the most preferred feature value. Comparing the total inference scores in blocks with *a* > *b* versus *b* > *c* evidence appearing last in a block (Figure 6), we find a marginal effect of trial order (*β* = −0.196, *SE* = 0.105, *t* = −1.86, *p* = 0.064)—a trend indicating that participants may have performed less well when they saw the information about the most preferred value early in the block (*b* > *c* blocks). In this analysis, we treated the total inference score as the dependent variable, the type of evidence block as an independent variable, and included random intercepts for participants.

Looking qualitatively at the errors, we see that after receiving *b* > *c* as the final piece of evidence, some participants rated the middle (*b*) value higher than the previously learned best preferred value (*a*), since the latter did not appear in the final trial. In other words, memory limitations may be responsible for less successful information integration in blocks where the information about the most preferred feature value came early in the block.

The difficulties in learning the hierarchy in the *a* > *b* > *c* blocks might have been additionally caused by a confound introduced by the wording of the task. The instructions specified that the listener always chooses her favorite feature value. These instructions were added to signal that the listener’s object choice is deterministic. Thus, “favorite” is predicted to be interpreted as favorite among the available options. The meaning of words is commonly restricted by the relevant context, or, to use linguistic terminology, by the current event or situation (Barwise & Perry, 1983; Kratzer, 2021). However, we cannot exclude the possibility that participants interpreted the predicate “favorite” as applying globally to the whole block of four trials, rather than to the current trial only. This interpretation would yield confusion when *b* > *c* evidence appeared last, as participants may have concluded that *b* was in fact the absolute favorite feature value.

#### Experiment 1: Summary.

In Experiment 1, we asked whether participants can a) infer choice priors of the listener upon observing how she resolves referential ambiguity and b) whether participants integrate information across a series of trials, manipulating the information that was available to the participant. We replicate the results of Achimova et al. (2022) and confirm that participants are indeed capable of inferring the choice priors of others upon observing a choice of an object in a situation where the utterance applies to more than one object. We further show that participants are more successful at integrating information across a series of trials for blocks with partial hierarchy—that is, blocks with less information to integrate. By analyzing the way that participants used trial evidence and the errors that resulted, it appears that errors are attributable to information integration recency effects and potentially misleading instructions.

## EXPERIMENT 2: COMBINED DESIGN

With evidence that speakers can use ambiguity-resolution behavior to infer choice priors in multi-shot signaling game scenarios, we next explore the utterance-selection behavior of speakers, seeing whether participants can strategically select ambiguous utterances in an attempt to learn about the choice priors of their listeners, and whether selecting ambiguous utterances leads to an increase in learning. In the process, we also explore whether the increased task engagement necessitated by utterance selection leads to better learning relative to Experiment 1, where participants encountered pre-selected utterances.

### Material and Methods

Experiment 2 featured a combined utterance-selection and choice-prior-inference design.^3^ On each trial, the participants first selected an utterance, then observed a choice of an object by the listener in response to the selected utterance, and then adjusted the sliders indicating the inferred listeners’ choice priors. The experiment was carried out on the Amazon Mechanical Turk crowd-sourcing platform.

#### Participants.

100 participants completed the experiment and received £1.5 as compensation. The experiment design and participant compensation were approved by the Pscyhology Department Ethics Committee at the University of Tübingen. We excluded data from two participants who self-identified as non-native speakers and from three other participants who reported that they were confused and did not fully understand the instructions. The experiment lasted approximately 9 minutes (*mean* = 9.3 minutes, 315 *median* = 8.8 minutes).

#### Design.

In each trial, the participants first selected an utterance and then watched a simulated listener choose an object. Participants completed 4 blocks of trials, each containing 4 trials. The simulated listener was kept constant within a block. Each subsequent block featured a different simulated listener. For the utterance-choice portion of the task, participants encountered combinations of objects that could potentially let the speakers infer the choice priors of the listener. Thus, we excluded scenarios with three identical objects, since no utterance can lead to learning about the choice priors with those objects. We also avoided scenarios with all objects being unique: if the objects do not share any properties, no utterance is ambiguous, and therefore no learning is possible.

**Figure 7.**
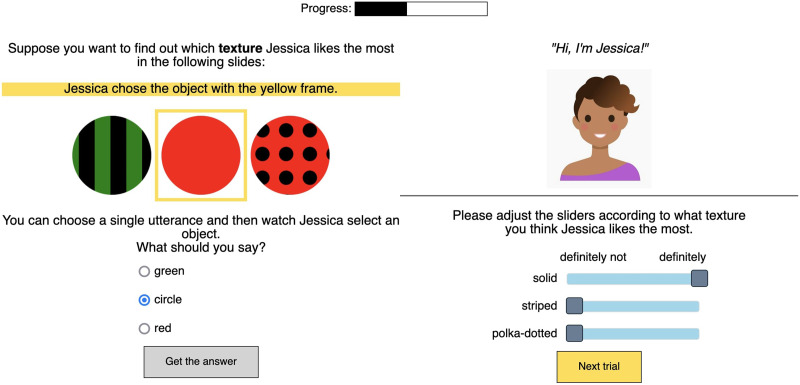
Sample trial for Experiment 2.

Once the participant selected an utterance, the simulated listener picked an object according to her implicit preferences, which always represent a full hierarchy: *a* > *b* > *c*. For example, if she preferred the solid texture to striped and polka-dotted in Figure 7, she would select a solid object if this choice was available. If solid objects did not appear in the scene, the listener would pick the next preferred object according to the implicit *a* > *b* > *c* hierarchy. This process was deterministic: the listener always picked an object with the most preferred feature value available given the current scene.

After observing the object choice, participants then adjusted sliders to indicate their beliefs about the listener’s choice priors. The information gain potential of this second part of the trial was modulated by the participants’ choices of an utterance in the first part. If participants chose ambiguous utterances that picked out multiple objects that differed in their target feature value, such a situation offered the potential for learning. However, if an unambiguous utterance was chosen, no choice priors of the listener could be learned because the object choice would be uninformative.

### Results

Unlike in Experiment 1, where we controlled the structure of blocks by either presenting the full information about the hierarchy (*a* > *b* > *c*) or only partial information (*a* > *b*, *c*) over the range of four trials, in Experiment 2 the type of learning scenario was determined by the participant’s utterance choices. Ambiguous informative utterances created learning opportunities, while unambiguous utterances did not permit any inferences. Despite the fact that we could not systematically manipulate the learning scenario (i.e., block type) as an experimental parameter, we were able to analyze the resulting trial configurations post-hoc. We identified the blocks where participants could have learned the full preference hierarchy *a* > *b* > *c* or the partial hierarchy *a* > *b*, *c*, given the utterances that they chose. The question we asked was whether they indeed succeeded in inferring the choice priors.

#### Inference success.

Just like in Experiment 1, we start by examining whether participants were more successful in inferring the preferred value *a* in *a* > *b*, *c* vs. *a* > *b* > *c* blocks. We again coded whether they identified the preferred value correctly at the end of each block as a binomial variable and treated the type of block as the independent variable. We then fit the data with a generalized linear mixed model with random intercepts for participants. The analysis revealed that, similarly to Experiment 1, participants were more successful in identifying the preferred value in the partial hierarchy blocks (*a* > *b*, *c*: mean score 0.831, *a* > *b* > *c*: mean score 0.722; *β* = 0.977, *SE* = 0.378, *z* = 2.580, *p* < 0.01). We also registered a significant interaction of experiment and type of block (*β* = −1.284, *SE* = 0.484, *z* = −2.651, *p* < 0.01): the performance was comparable in *a* > *b*, *c* blocks over 2 experiments (*mean*_1_ = 0.86; *mean*_2_ = 0.83), while the the scores diverged to a greater extent between experiments in *a* > *b* > *c* blocks (*mean*_1_ = 0.5; *mean*_2_ = 0.72). Overall, participants were more successful in identifying the preferred value in Experiment 2 (*β* = 1.048, *SE* = 0.325, *z* = 3.220, *p* < 0.01).

#### Integration of evidence across trials.

**Figure 8.**
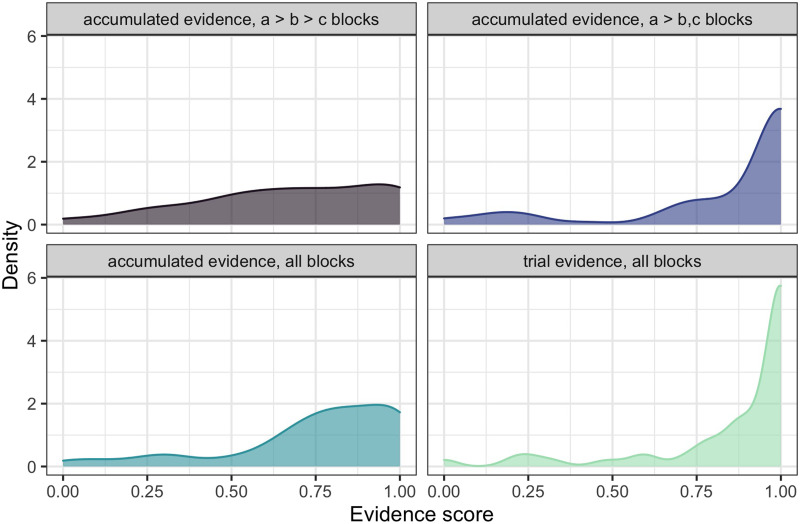
**Experiment 2: Density plots over the proportion of evidence-respecting preference inference trials, dependent on the available trial evidence (right bottom) or accumulated evidence in all blocks (bottom left) or block-respective (top panels).** Compared with Experiment 1 (cf. Figure 5), the inferred accumulated information is of higher quality.

Next, we look at participants’ ability to infer the full hierarchy of preferences, looking at accumulated evidence scores which signal whether participants used the available evidence from the previous trials to update their inference of the listener’s choice priors. Figure 8 plots the distribution of participants’ accumulated evidence scores and the trial evidence score (bottom right panel). Unlike in the analysis reported above where we focused on inferring the preferred value at the end of the block, here we use all the trials of each block. We analyze whether the hierarchy that participants indicate at each step corresponds to the information about choice priors that was in principle available to them.

The density plots in Figure 8 illustrate that participants were more successful in integrating evidence in the *a* > *b*, *c* blocks compared to the *a* > *b* > *c* blocks: the purple distribution in the top right is skewed to the right, while the gray distribution in the top left distributes the density mass more evenly. This interpretation is confirmed by the results of a generalized mixed effects model. Our model predicted binary accumulated evidence scores by the type of block; we also fit random intercepts for participants. The analysis reveals that, as in Experiment 1, participants integrated evidence more successfully in *a* > *b*, *c* blocks (*mean* = 0.837) compared to full hierarchy *a* > *b* > *c* blocks (*mean* = 0.557; *β* = 2.843, *SE* = 0.328, *z* = 8.658, *p* < 0.001).

#### Analysis of errors.

**Figure 9.**
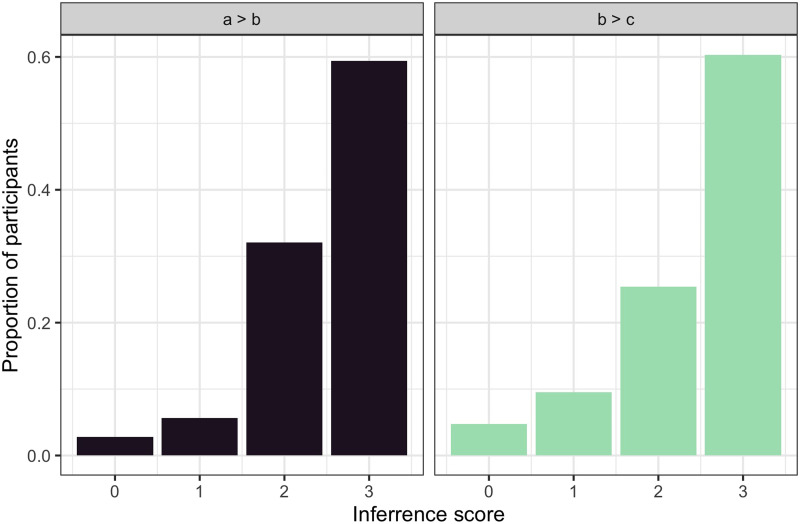
**Experiment 2: Histograms of total inference scores for the blocks in which the full *a* > *b* > *c* preference hierarchy could be learned, dependent on whether information about *a* > *b* or *b* > *c* was available in the last trial(s).** In contrast to Experiment 1 (cf. Figure 6), the two densities hardly differ, indicating a lower recency bias and thus a better accumulative integration of information.

In Figure 9, we look at the total inference scores for *a* > *b* > *c* blocks depending on whether the *a* > *b* or *b* > *c* information was elicited later in the trials. We calculate total inference scores by evaluating which of the pairs from the hierarchy *a* > *b* > *c* were identified correctly. For each of the pairs {a, b}, {b, c}, and {a, c}, we assign a value of 1 and then sum them up. The first thing to note is that these inference scores are overall higher for Experiment 2 compared to Experiment 1: they are skewed to the right regardless of which piece of evidence came later, with almost 60% of participants inferring the correct full hierarchy when experiencing *a* > *b* > *c* blocks. To assess this effect quantitatively, we fit the data with a linear mixed model, treating the inference score as the dependent variable, and the experiment (1 vs. 2) as an independent variable. The random effect structure included random intercepts for participants. The analysis revealed that participants achieved higher inference scores in *a* > *b* > *c* blocks for Experiment 2 (*mean* = 2.15) compared to Experiment 1 (*mean* = 1.94) (*β* = 0.216, *SE* = 0.08, *t* = 2.418, *p* = 0.017).

#### Strategic ambiguity.

We hypothesized that learning success (i.e., was the full choice prior hierarchy inferred?) depends on the quality of utterances that the participants selected. We define utterance quality in a trial based on a three-step procedure. First, we identify whether the utterance that a person selected is ambiguous; in other words, does the utterance apply to more than one object? Second, we check the extent to which the utterance-conforming objects differ on the target feature dimension. For example, if the speaker selected the utterance “red” and there are two red objects in the scene, we check whether they differ in their target feature values (if the target feature is “shape”, we check whether they are both clouds, circles, or squares). If the utterance “red” picks out a red circle and a red square, it receives a score of 2; if “red” applies to a red circle, a red square, and a red cloud, it receives a score of 3. Unambiguous utterances receive the score 1. Third, we evaluate how the utterance compares to the best possible utterance in that trial. This comparison transforms the score calculated in the previous step into a value between 0 (worst) and 1 (best). This transformed value is the utterance quality score. The utterance quality score reflects whether a person chose an utterance that was ambiguous (applied to more than 1 object), informative (it applied to objects that differ on the target dimension), and optimal (there is no other utterance that would allow learning about more target feature values).

**Figure 10.**
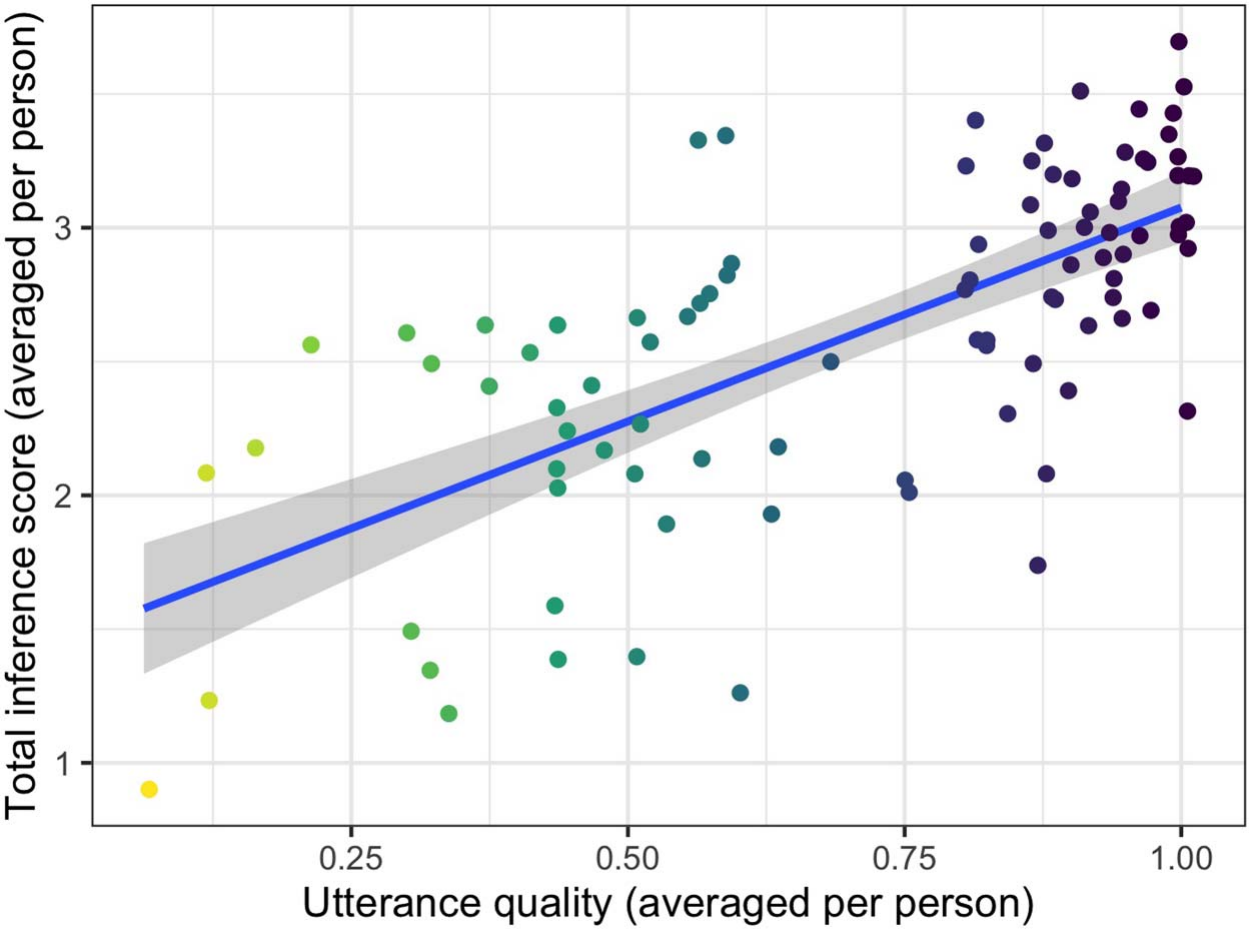
**Experiment 2: Utterance quality correlates with the total inference scores.** Thus, more ambiguous utterances indeed enable participants to learn more about the hidden preference hierarchy in our task.

We can now evaluate whether the utterance quality score is a predictor of the overall performance in the inference task. To get a metric of the performance, we assess whether participants inferred the full hierarchy of preferences *a* > *b* > *c*. Like in Experiment 1, we assign a score of 1 for every relation between values inferred correctly. For example, a participant who inferred the relations *a* > *b*, *b* > *c*, *a* > *c* receives the **total inference score** of 3. To plot the total inference scores against utterances quality scores, we first calculated average scores for every person depending on the trials that they saw in the experiment. Figure 10 plots average inference scores against average utterance quality scores, showing that when participants strategically selected more ambiguous utterances that created the potential for learning, they indeed were more likely to learn the choice priors better; this result is confirmed by a linear model where we treated total inference scores averaged per person as the dependent variable and the corresponding utterance quality scores as the independent variable (*β* = 1.599, *SE* = 0.1752, *t* = 9.124, *p* < 0.001). Utterance quality explains 46% of variance in the learning scores.

**Figure 11.**
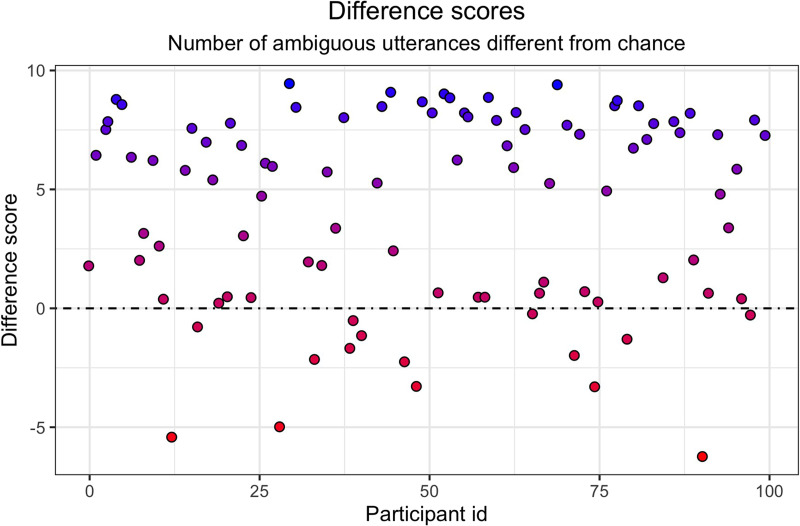
**Experiment 2: Difference scores by participant.** The difference score measures strategic usage of ambiguity. Values below zero imply active ambiguity avoidance, while values significantly above zero imply strategic ambiguity choices.

The utterance quality scores we calculated above depends on two interrelated properties of the utterances: their ambiguity and their informativity. When calculating the utterance quality score, we rewarded the choice of ambiguous utterances. However, since all trials contained at least one ambiguous utterance, it is possible that participants picked ambiguous utterances by chance rather than strategically. In order to assess the strategic aspect of utterance choice, we calculated the chance level of ambiguous utterances for each participant depending on the trials they saw, and then subtracted the number of ambiguous utterances predicted by chance from the number of ambiguous utterances each participant selected. Figure 11 shows the resulting difference scores: it plots participant IDs on the *x*-axis and their difference scores on the *y*-axis. Color coding reflects the magnitude and the polarity of the score. We observe that, while some participants strategically chose non-ambiguous utterances (data points below the reference line), 84% of the datapoints fall above the reference line, and darker color coding marks those participants who strategically and systematically chose ambiguous utterances. As a conservative estimate of the proportion of participants who strategically chose ambiguous utterances, we see that 55% have difference scores above 5.

#### Experiment 2: Summary.

In sum, higher rates of selecting informative ambiguous utterances are associated with greater success in learning the full hierarchy of choice priors of the listener, as demonstrated by the comparison of inference scores across experiments. Moreover, a group of participants chooses ambiguous utterances systematically rather than randomly, suggesting that they are strategically choosing those utterances to improve their learning chances.

## GENERAL DISCUSSION

In this paper, we focused on the factors that determine the success of choice prior inference in a situation of observing a referential choice. We have demonstrated that participants are capable of inferring not only simple priors upon observing a single act of disambiguation, but also more complex, hierarchical choice priors by accumulating evidence over multiple trials in a rational manner. Experiment 1 revealed that, despite the low overall number of trials (only four trials in each block), many participants managed to successfully integrate the available information about preferences. This process was easier when only a simple *a* > *b*, *c* feature hierarchy had to be learned. The fact that redundant information about the simulated choice priors helped to get the choice prior hierarchy correct indicates that the task was quite challenging. A deeper analysis of those cases where participants failed to infer the relevant hierarchy showed that some participants exhibited a recency bias—perhaps driven by the task instructions—which led to overwriting previously encountered pair-wise choice prior differences: participants performed better at correctly concluding that *a* was the highest ranked option among the relevant choices in the choice prior in blocks of trials where *a* > *b* information came last compared to blocks that featured *b* > *c* trials as the last ones. Overall, the results of our first experiment imply that iterative evidence accumulation is possible but challenging in the investigated signaling game scenario, perhaps because the scenario is somewhat artificial, but also because our instructions may have been misleading. We thus moved on to a more active social interaction scenario in Experiment 2.

Experiment 2 demonstrated that being able to play an active part in generating learning scenarios—and thereby presumably being more engaged in the task—yielded higher inference success. The data also revealed that the use of ambiguous utterances in the signaling game scenario indeed allowed for learning about the listener’s priors. We observed that participants were more likely to infer the correct priors if they used informative ambiguous utterances, confirming that they are capable of strategically employing ambiguity as an epistemic tool. Moreover, our results suggest that the observation of the full signaling game, including the active utterance choice, helps participants to make use of the full Bayesian inference pipeline. This conclusion is supported by the fact that the recency bias was much smaller in Experiment 2 compared to Experiment 1, particularly in the participants that managed to choose utterances in a way that yielded sufficient information to extract the full *a* > *b* > *c* choice prior hierarchy. Thus, Experiment 2 showed that task engagement can be enhanced when full signaling games are played out pragmatically. Moreover, it confirms that more complex hierarchies can be learned iteratively over time by corroborating choice information over successive trials. In the future, we plan to model the observed behavior by means of Achimova et al.’s (2022) RSA-based utterance choice and choice prior-inference mechanisms, potentially contrasting iterative Bayesian updating with reinforcement learning approaches (Ciranka et al., 2022; Glasauer, 2019).

Despite the apparent simplicity of our task, we observed that memory limitations in some circumstances likely prevented the successful integration of choice priors. Unlike in one of the experiments reported in Baker et al. (2017), our participants received no prior information about the structure of the relevant choice priors. Baker et al. informed their participants that the agent preferred property *a* over property *b*, and properties *a* and *b* over property *c*, thus providing prior expectations that might structure the inference process. The absence of this information in our experiments might be partially responsible for lower inference scores for the full choice prior hierarchy, particularly in Experiment 1 without active speaker engagement. However, in Experiment 2 we show that enabling participants to set up their experiment themselves, thus actively creating ambiguous instructive choice situations and observing the ambiguity resolution behavior, facilitates choice prior inference. With at least 55% of participants systematically selecting ambiguous utterances (their difference rate was above 5 in Figure 11), it appears that the multi-trial utterance-choice setting of our current task yields greater rates of ambiguous utterance selection than the 26% of participants identified as having done so in the single-trial experiment of Achimova et al. (2022).^4^ This comparison indicates that the observation of the full signaling game trials may have better motivated the potential utility of ambiguity. When participants could observe the listener’s choice of an object following the utterance they selected, they were able to better anticipate in subsequent trials what types of utterances are useful for learning. Compared to Experiment 1, consequent choice prior inference was more successful, further supporting the benefit of setting up actual inference experiments. While the participants did make inference errors in integrating the information across a series of trials, their performance nevertheless remains relatively high given the number of trials that were available. Experiments that target the inference of more complex choice priors that include seven rather than three values of the target feature and involve transitive inference can include as many as 300–500 evidence trials (Ciranka et al., 2022).

Despite the observed increase in strategic utterance choices, our results still confirm that actively engineering learning opportunities remains a complex task for some participants. In this paper, we used the case of referential ambiguity to create a situation where a behavioral choice may reflect the person’s priors. A further look into strategic ambiguity as a linguistic phenomenon may in fact suggest a possible source of how such learning opportunities develop. Theoretical models of dog-whistles (Henderson & McCready, 2017) and strategic indirectness (Pinker et al., 2008) suggest that ambiguity may emerge as an epiphenomenon of the speaker simultaneously pursuing a combination of information transfer and social goals. More recent experimental evidence suggests that indirectness can also emerge when speakers optimize social goals along with information transfer goals (Yoon et al., 2020). Independently of how referential choices emerge, the listener’s response reveals aspects of her choice prior. The results presented here suggest that active engagement and iterative social exchanges can increase the chance of inference success. Whether this is the case also in more natural social interactions remains to be shown.

## DATA AND MATERIALS AVAILABILITY

Data and analysis code are available at this OSF repository: https://osf.io/yn4wd/?view_only=a723e0e89688475ea022cf59d2e3e9df.

## ACKNOWLEDGMENTS

We would like to thank Johannes Bertram (University of Tübingen) for his help with experiment implementation, data processing and analysis. Two anonymous reviewers provided thoughtful comments and suggestions and allowed us to see the results in a new light.

## FUNDING INFORMATION

The project was funded by the Deutsche Forschungsgemeinschaft (DFG, German Research Foundation) via the Research Training Group 1808: Ambiguity - Production and Perception, project number 198647426. Martin V. Butz is also a member of the Machine Learning Cluster of Excellence, EXC number 2064/1 – Project number 390727645.

## AUTHOR CONTRIBUTIONS

Asya Achimova: Conceptualization: Equal; Data curation: Lead; Formal analysis: Lead; Methodology: Equal; Visualization: Lead; Writing – Original draft: Lead. Gregory Scontras: Conceptualization: Equal; Formal analysis: Supporting; Visualization: Supporting; Writing – Original draft: Equal. Ella Eisemann: Conceptualization: Equal; Data curation: Equal; Methodology: Equal; Visualization: Supporting; Writing – Original draft: Supporting. Martin V. Butz: Conceptualization: Equal; Formal analysis: Supporting; Funding acquisition: Lead; Methodology: Equal; Supervision: Lead; Visualization: Supporting; Writing – Original draft: Equal.

## Notes

^1^ See Achimova et al. (2022) for a full discussion of the variability in utterance choice strategies across participants and across several experiments.^2^ Experiment 1 is available at https://cognitive-modeling-experiments.uni-tuebingen.de/publix/10/start?batchId=17&generalMultiple.^3^ Experiment 2 is available at https://cognitive-modeling-experiments.uni-tuebingen.de/publix/9/start?batchId=18&generalMultiple.^4^ It is important to acknowledge that the procedures for calculating the proportion of participants who use ambiguity strategically is not identical across the two studies. Achimova et al. (2022) used a modeling approach and identified “strategic” participants based on the value of a parameter that regulates the choice of utterances by scaling how important information gain is for a given person. In the present work, we conservatively identify strategic ambiguity use by assessing whether a participant chose ambiguous utterances markedly more often than would be expected by chance. However, in both cases, the selection of a cut-off point that separates strategic ambiguity from its non-strategic use is to a certain degree arbitrary.
